# Calcium-Activated Potassium Channels in Ischemia–Reperfusion: Learning for the Clinical Application

**DOI:** 10.3389/fmed.2015.00021

**Published:** 2015-04-08

**Authors:** Mohamed S. A. Mohamed

**Affiliations:** ^1^Thoracic Transplantation Department, University Clinic Essen, Essen, Germany

**Keywords:** *ex vivo* lung perfusion, lung transplantation, ischemic–reperfusion injury, Ca^++^-activated K^+^ channels, inflammatory cytokines, primary graft dysfunction

The article by Tano and Gollasch published in Frontiers in Physiology reviewed the involvement of Ca^++^-sensitive K^+^ channels in ischemia and reperfusion, with cardio-vascular and brain models mostly discussed (1), where the increased Na^+^ inflow activates Na^+^–Ca^++^ exchanger, and leads to cell membrane depolarization (2). Activated Na^+^–Ca^++^ exchanger works to pump Na^+^ out and Ca^++^ in (2). The increase in intracellular Ca^++^ results in activation of various Ca^++^-sensitive K^+^ channels to establish K^+^ influx and hyperpolarization (1).

In the scenario of *lung* transplantation, the graft is subjected to ischemia followed by reperfusion (following standard transplantation or during *ex vivo* perfusion). Graft ischemia results in inhibition of Na^+^–K^+^ ATPase, inhibition of K^+^ ATP channels, drop of intracellular K^+^, and the absence of flow favors cell membrane depolarization (2). Cell membrane depolarization and inactive K^+^ ATP channels would be associated with increased NADPH oxidase (NOX2) activity and increased production of reactive oxygen species (ROS) (2). ROS results in inflammasomes priming (3). Decreased intracellular K^+^ results in inflammasomes activation. Inflammasomes activation results in caspase 1 activation, which activates pro IL1β and pro IL18 (3). Both IL1β and IL18 are able to induce IL6.

Accordingly, the enhancement of Ca^++^-sensitive K^+^ channels during lung graft ischemia would be expected to provide protection through antagonizing membrane depolarization (i.e., favoring hyperpolarization), which would attenuate ROS production, leading to abortion of inflammasomes priming and activation, and accordingly the release of pro-inflammatory cytokines.

The Toronto team of lung transplantation has achieved significant inhibition of cytokines production within the lung graft through gene therapy during *ex vivo* lung perfusion (adenoviral IL10 delivery), which correlated with decreased incidence of primary graft dysfunction and chronic lung allograft dysfunction after transplantation (4). However, another study reported similar level of inhibited cytokines production through inhalation of 2% hydrogen. This was achieved through the up-regulation of hemeoxygenase-1 (HO-1) (5). HO-1 catalyzes the production of carbon monoxide, which activates big conductance Ca^++^-activated K^+^ channels (6).

These findings highlight the possible protective role of the enhancement of Ca^++^-activated K^+^ channels during lung graft ischemia. Accordingly, further studies should be conducted to investigate the actual status of these channels during lung graft ischemia prior to transplantation. In addition, pharmacological activation of these channels could be a good target to protect the lung graft during transplantation, with corresponding improvement of the clinical outcome.

## Conflict of Interest Statement

The author declares that the research was conducted in the absence of any commercial or financial relationships that could be construed as a potential conflict of interest.
